# Supplementary material for: Human norovirus targets enteroendocrine epithelial cells in the small intestine
Source: Nat Commun. 2020 Jun 2;11:2759. doi: 10.1038/s41467-020-16491-3 (PMC7265440; doi:10.1038/s41467-020-16491-3)
Supplement: Supplementary file 1 — Supplementary Information [file 41467_2020_16491_MOESM1_ESM.pdf]

Supplementary Information

Human Norovirus Targets Enteroendocrine Epithelial Cells in the Small Intestine

Green et al.

This file contains:
Supplementary Figures 1-13
Supplementary Tables 1 and 2

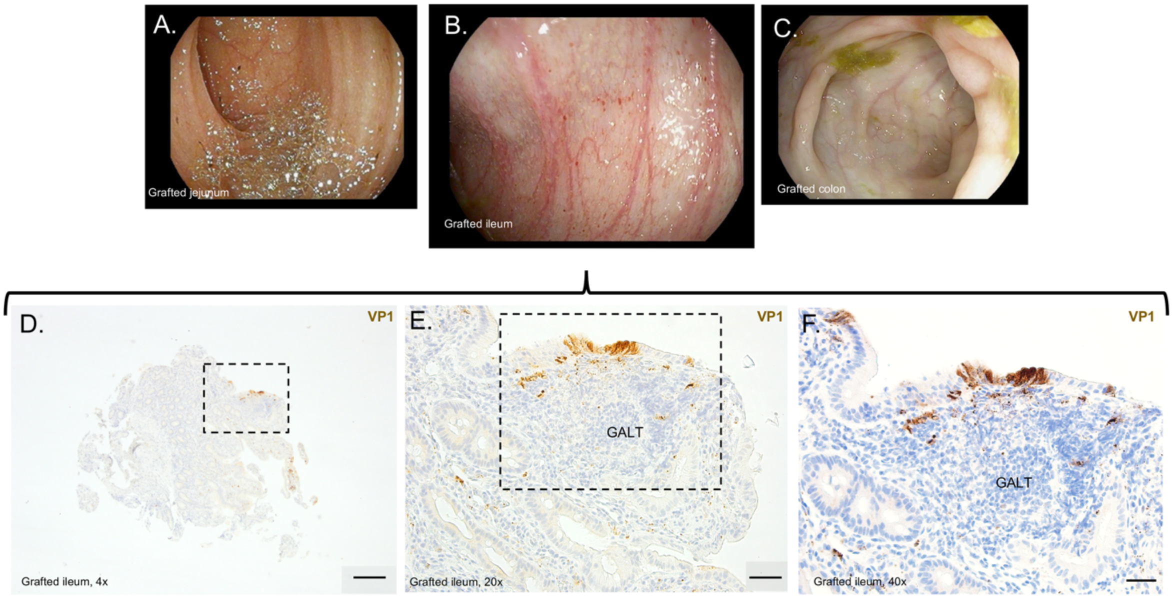

Supplementary Figure 1: Gastrointestinal pan-endoscopy imaging and biopsy analysis of patient GT-1. Endoscopic images of enteric tract during acute norovirus infection showing: **A.** Upper grafted jejunum **B.** Grafted ileum with areas of mucosal petechiation and **C.** Grafted colon. A norovirus-positive area of an ileal biopsy was visualized by IHC and chromogenic staining with capsid-specific monoclonal antibody TV19 and is shown at increasing magnifications of: **D.** 4x **E.** 20x and **F.** 40x; scale bars represent 200μm, 50μm and 20μm, respectively.

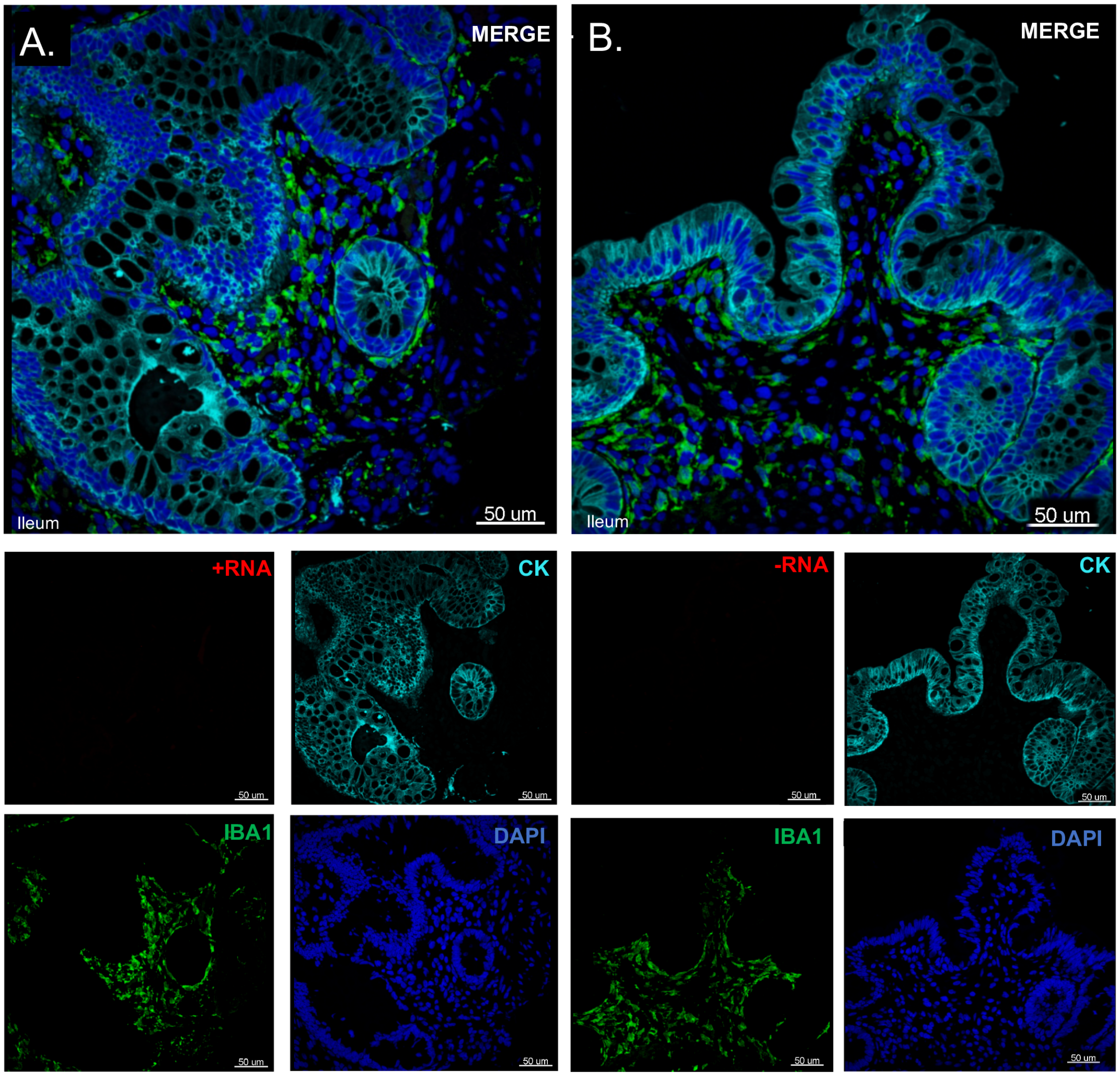

Supplementary Figure 2: Norovirus-negative tissue control for the RNA probes in this study. An ileal biopsy section from a norovirus-negative individual (GT-5) was hybridized with RNAscope probes (that would appear red) for the detection of: **A.** Positive or **B.** Negative sense norovirus RNA. Norovirus RNA signals were not detected in norovirus-negative biopsies, as represented in the images from this patient. Markers cytokeratin (cyan) and macrophage protein IBA-1 (green) were used to localize the epithelial layer and lamina propria, respectively. Nuclei were stained with DAPI (blue).

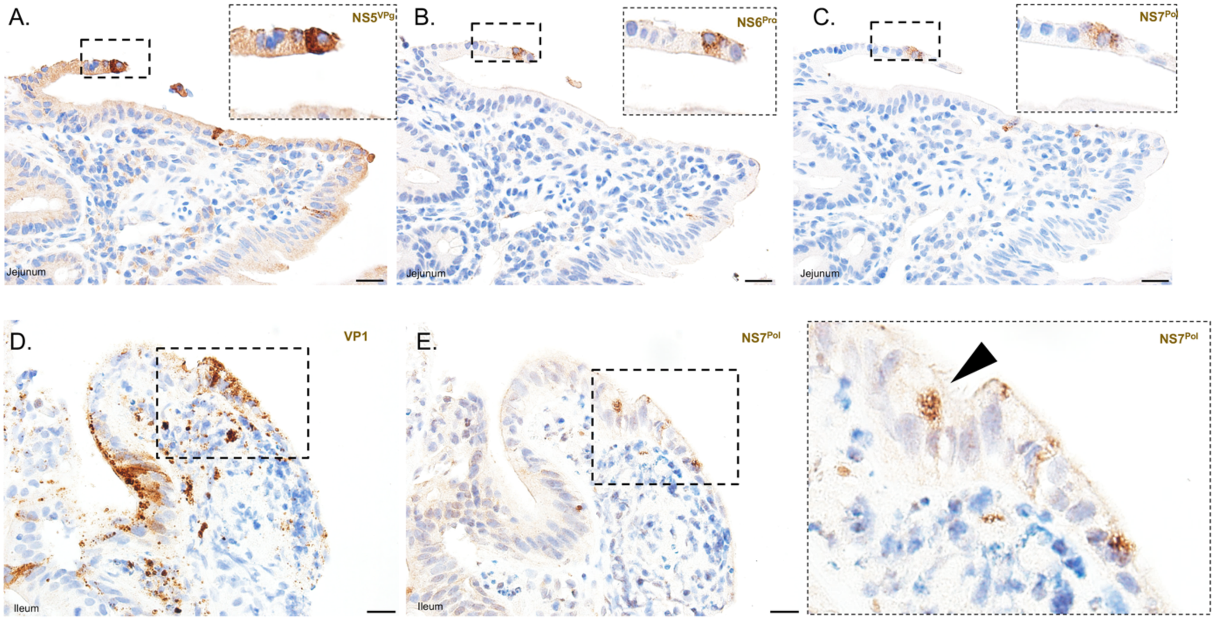

Supplementary Figure 3: Detection of norovirus nonstructural proteins in patient GT-1 jejunal biopsies. Sections from a jejunal biopsy were probed with hyperimmune sera raised against norovirus GII.4 nonstructural proteins: **A.** $NS5^{VPg}$, **B.** $NS6^{Pro}$, and **C.** $NS7^{Pol}$ A magnified inset in each panel shows the morphology of a selected epithelial cell strongly positive for all three nonstructural proteins, consistent with active viral replication. Differences in the efficiency of detection between the capsid monoclonal antibody and the nonstructural polymerase protein ($NS7^{Pol}$) hyperimmune serum are shown in **D.** and **E.**, respectively. Inset in Panel **E.** shows a magnified view of viral RNA-dependent RNA polymerase (NS7) expression, with arrow highlighting positive cell. Magnification **A-E**: 40x, scale bars represent 20μm.

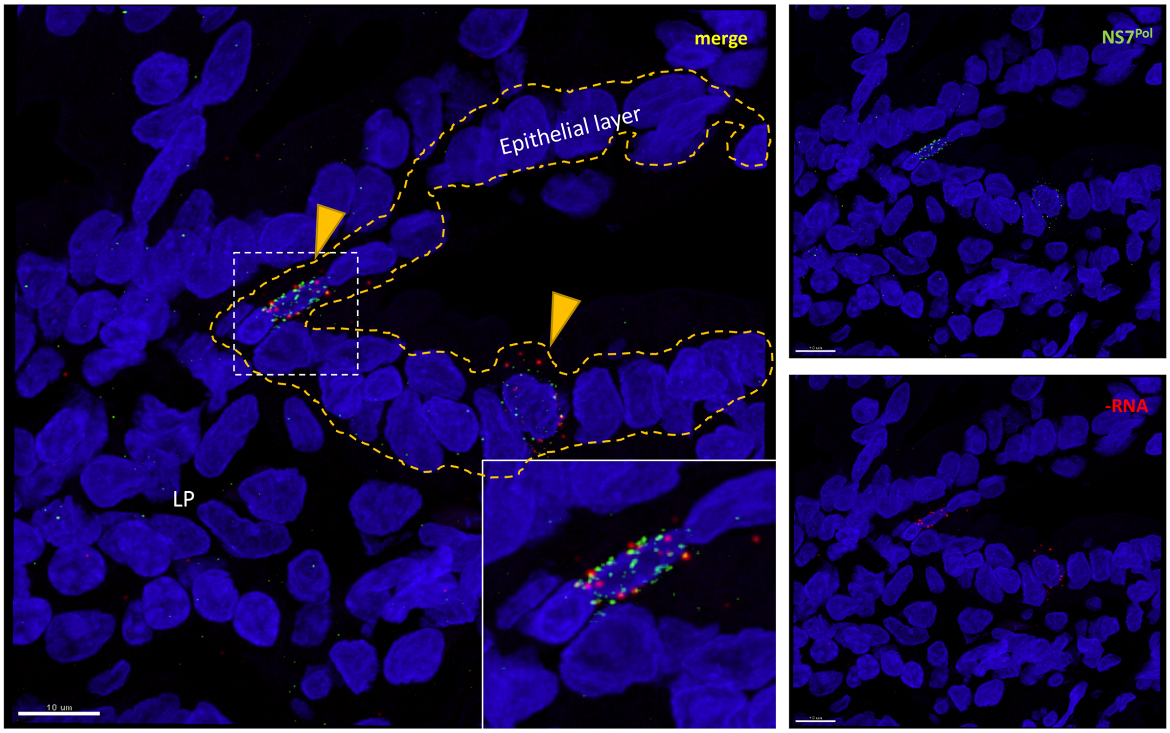

Supplementary Figure 4: Visualization of two epithelial cells expressing both NS7^{Pol} and negative sense norovirus RNA. Confocal microscopy imaging of NS7^{Pol} (green, detected with hyperimmune serum raised against recombinant NS7^{Pol}) and negative sense RNA (red, visualized with RNAscope probe) was performed on the jejunal biopsy of patient GT-1. The epithelial layer (based on morphology) is outlined with a dotted orange line and a region consistent with lamina propria is labeled as LP. Epithelial cells expressing both signals are indicated with orange arrows in the merged image. Inset shows magnified view of the upper cell. Nuclei were stained with DAPI (blue).

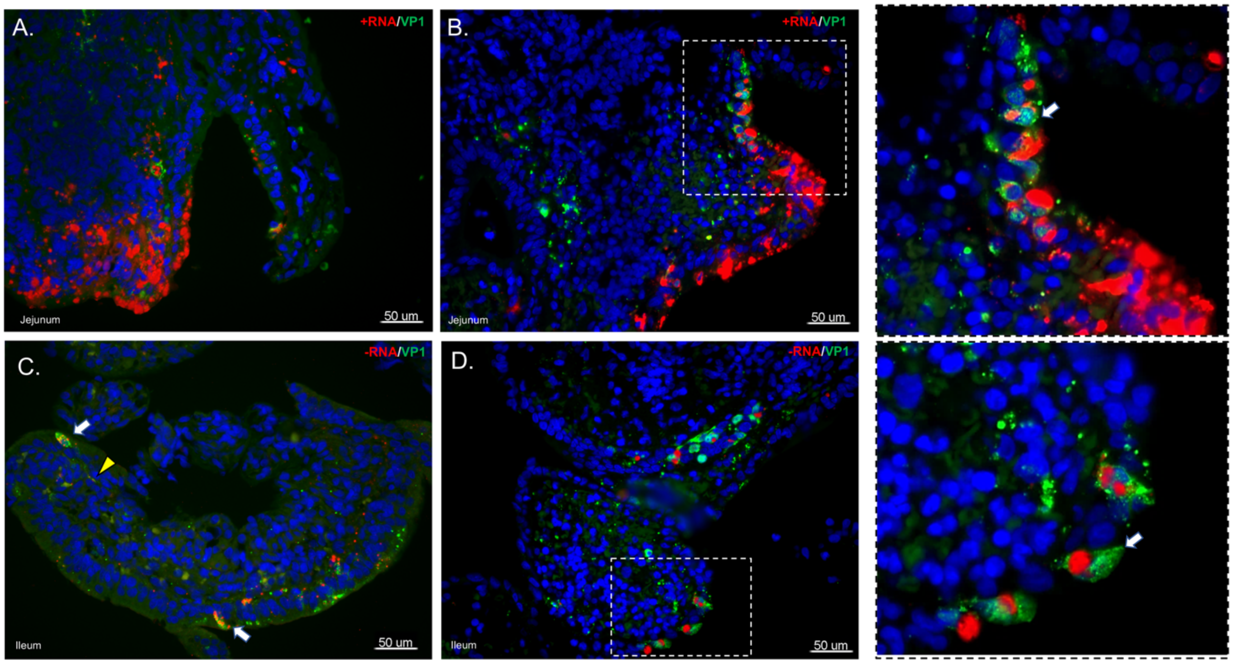

Supplementary Figure 5: Association of VP1 expression with positive or negative sense norovirus RNA. Jejunal and ileal biopsies of patient GT-1 were probed to assess the general patterns of RNA and capsid protein distribution in tissue in the absence of cellular markers. Nuclei were stained with DAPI (blue). **A. and B.** Fluorescence microscopy imaging of VP1 (green, visualized with TV19) and positive sense RNA (red, visualized with RNAscope probe) in jejunum. Inset in panel **B**. shows magnified view of villous tip area where both positive sense RNA and capsid protein were expressed within the same cell (white arrow). Strong positive strand RNA signals were observed with the RNAscope probe, which has an inherent signal amplifying feature. **C.** Fluorescence microscopy imaging of VP1 (green, visualized with TV19) and negative sense RNA (red, visualized with RNAscope probe) in ileum. Certain cells (marked with white arrows) contained both negative sense RNA and capsid protein and were spatially and morphologically consistent with epithelial cells. Scattered negative sense RNA and capsid protein were observed within the subepithelial regions in this biopsy section (yellow arrow), but the associated cell type was not clear in the absence of cellular markers. **D.** Fluorescence microscopy imaging of VP1 (green, visualized with TV19) and negative sense RNA (red, visualized with RNAscope probe) in ileal tissue. White arrow in inset shows cell containing both negative sense RNA and VP1.

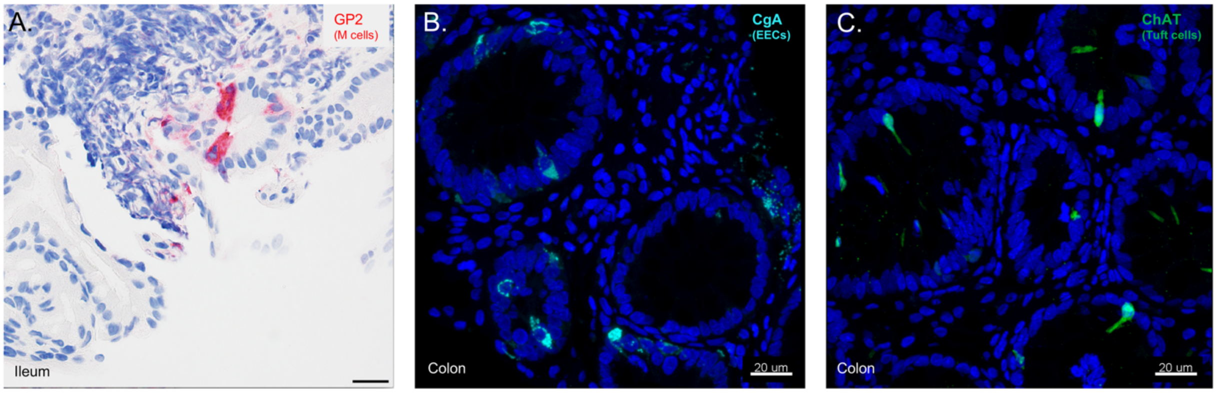

Supplementary Figure 6: Performance of antibodies to detect specialized epithelial cell markers in intestinal biopsy tissue from patient GT-1. The following markers were chosen for analysis in this study: **A.** Glycoprotein 2 (GP2) **B.** Chromogranin A (CgA) and **C.** Choline acetyltransferase (ChAT) as optimal for the identification of M cells, enteroendocrine cells (EECs) and tuft cells, respectively. Magnification **A-C**: 40x, scale bars represent 20µm.

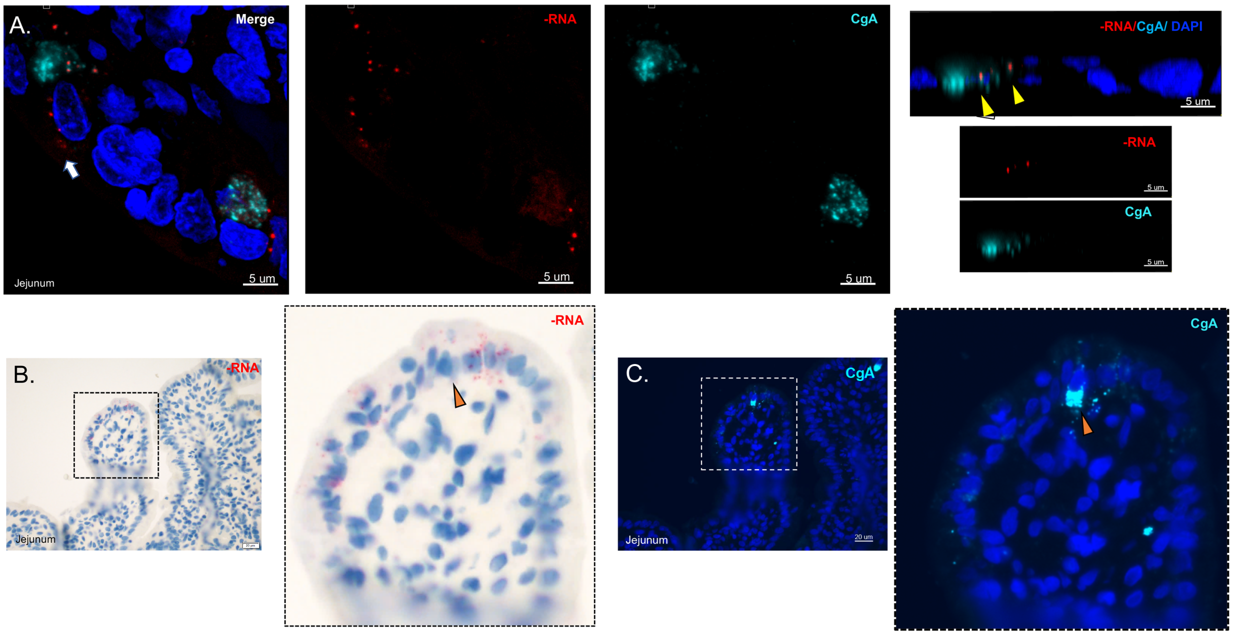

Supplementary Figure 7: Relationship of norovirus negative sense RNA to chromogranin A (CgA)-positive and CgA-negative epithelial cells. A. Confocal imaging of two CgA-positive cells (cyan) and one CgA-negative epithelial cell with negative sense norovirus RNA (red) detected by *in situ* hybridization. Cross-section in far-right panel with yellow arrows shows intracellular location of CgA and negative strand RNA. Note the CgA-negative epithelial cell in first panel containing norovirus negative strand RNA (white arrow). **B. and C.** A section of the jejunum was visualized by brightfield microscopy following *in situ* hybridization with the negative sense probe (red). The same section was then incubated with antibodies to CgA (cyan) for visualization of EECs by immunofluorescence microscopy. A CgA-positive cell that did not bear evidence of norovirus infection is indicated by orange arrow in the corresponding inset for each panel. Magnification **B** and **C**: 40x, scale bar represents 20μm.

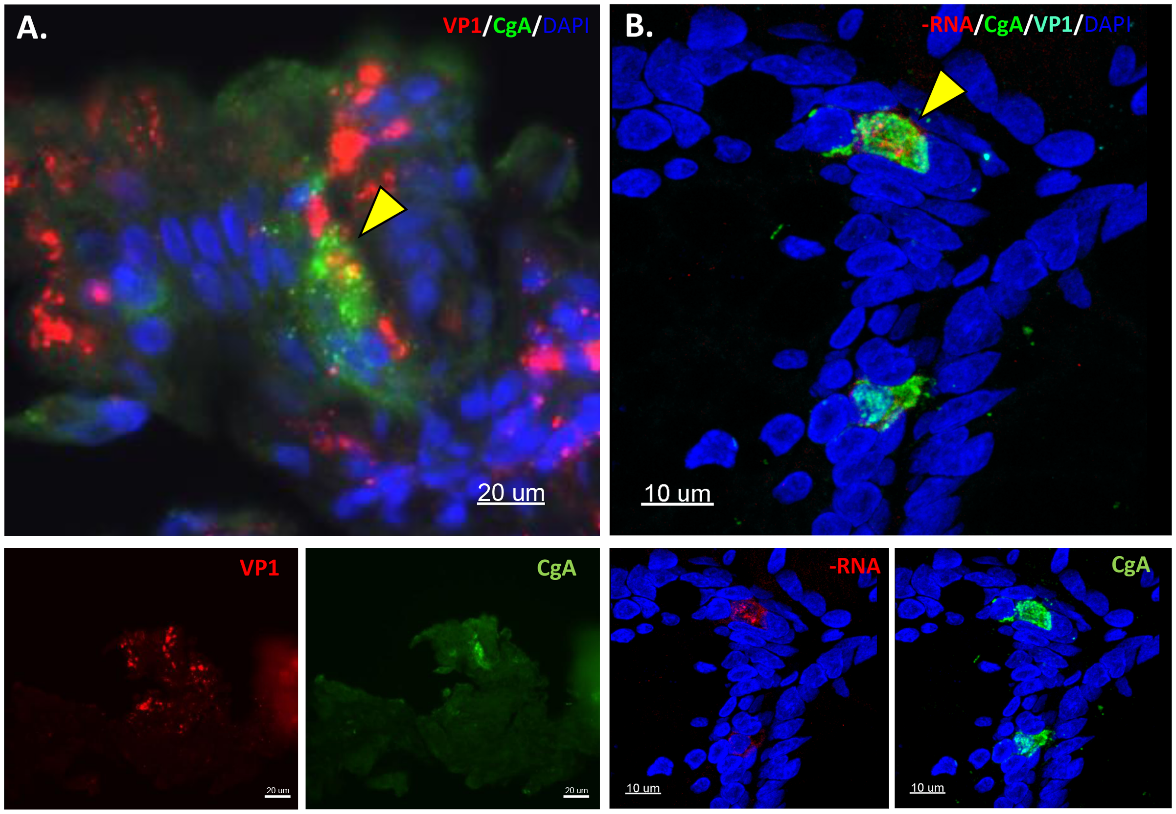

Supplementary Figure 8: Association of norovirus infection with enteroendocrine cells (EECs) in two additional patients. Additional intestinal biopsies were tested to determine whether EECs might be targeted in other individuals. **A**. Immunofluorescence imaging of intestinal biopsy from a second pediatric intestinal transplant recipient (GT-1211) who developed acute norovirus diarrhea in 2008. Stool was not available for genotyping of the strain. Tissue was probed with cross-reactive norovirus VP1 monoclonal antibody TV19 (red) and anti-CgA (green). Yellow arrow highlights a cell positive for both antibodies. **B.** Confocal imaging analysis of duodenal tissue from an adult patient (NIH-76) who was immunosuppressed post stem cell transplant and chronically infected with GII.4 Sydney[P16] norovirus. The GII.4-specific RNAscope probe developed in this study hybridized with negative sense norovirus RNA (red) in a subset of CgA-positive cells (green), as illustrated by the cell marked with a yellow arrow. Magnifications: **A** 40x; **B** 63x.

A. Sample ID	Intestinal region	Ratio infected EEC/non-infected EEC in 3 fields (percent)	Field magnification
**Patient NIH-74**	Duodenum	1/14 (7); 1/6 (16.6); 2/12 (16.6)	40x
**Patient GT-1**	Grafted Jejunum	1/10 (10); 1/26 (3.8); 1/28 (3.5)	40x
**Patient NIH-76**	Ascending colon	2/19 (10.5); 1/7 (14.2); 1/4 (25)	40x

B.

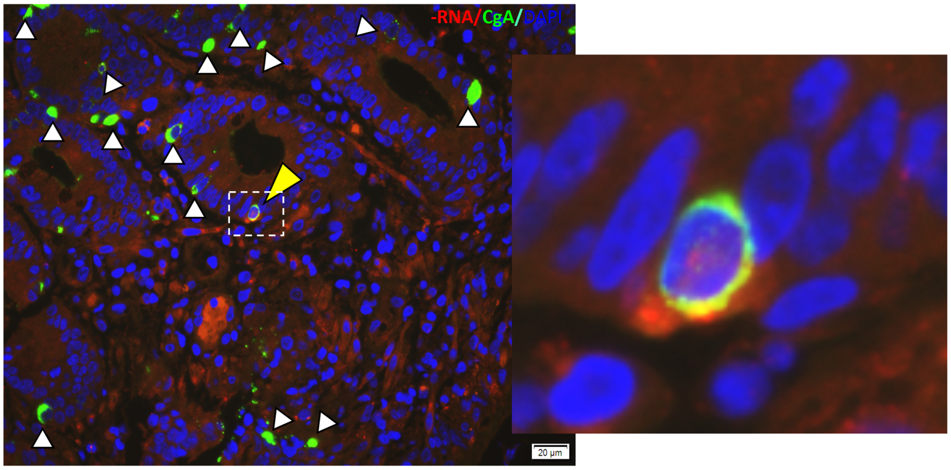

Supplementary Figure 9: Quantification of norovirus-infected enteroendocrine cells (EECs) in three patients. Patient GT-1 was described in this study. Patients NIH-74 and NIH-76 were adult individuals enrolled in NIH clinical protocols who were immunosuppressed post stem cell transplantation, and chronically infected with GII.4 Sydney[P31] and GII.4 Sydney[P16], respectively. Norovirus-infected EECs in human intestinal biopsy sections were evaluated at 40x magnification. Within the 40x field of view, cells were counted if they were positive for chromogranin A (CgA) alone (appearing green) or positive for both CgA and norovirus negative sense RNA (appearing yellow). **A.** Table summarizes the ratios of norovirus-infected versus non-infected EECs visualized at 40x magnification in three random fields for each biopsy specimen. Conversion of each ratio to percent showed a wide range from 3.5 to 25% positive EECs among fields of view, with an overall average of approximately 12%. Host and tissue variation, as well as the precise quantitation and identity of susceptible EEC subtypes will require further study. **B**. Representative field of view used in analysis. At least one norovirus-positive EEC was seen in most fields and a representative field for the ascending colon biopsy of NIH patient 76 is shown. White arrows highlight cells that were CgA-positive only. Yellow arrow highlights a cell appearing yellow in the field of view that is positive for both CgA (green) and norovirus negative sense RNA (red).

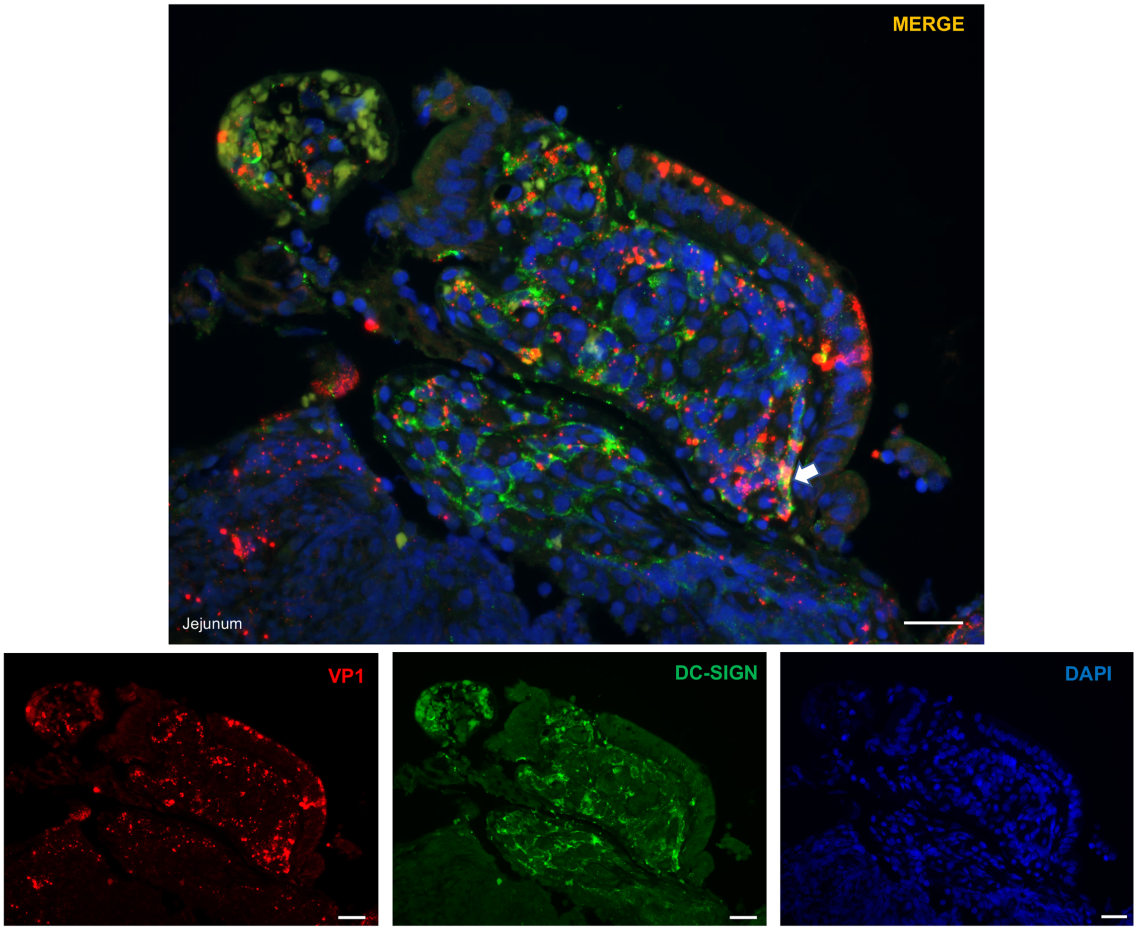

Supplementary Figure 10: Norovirus VP1 proteins co-localize with DC-SIGN positive cells in the lamina propria. The GT-1 biopsy was probed to detect VP1 with mAb TV19 (red) and DC-SIGN (green). Confocal imaging verified the presence of capsid protein in DC-SIGN bearing antigen presenting cells in the merged image (yellow) and illustrated with white arrow. Magnification: 40x, scale bar represents 20μm.

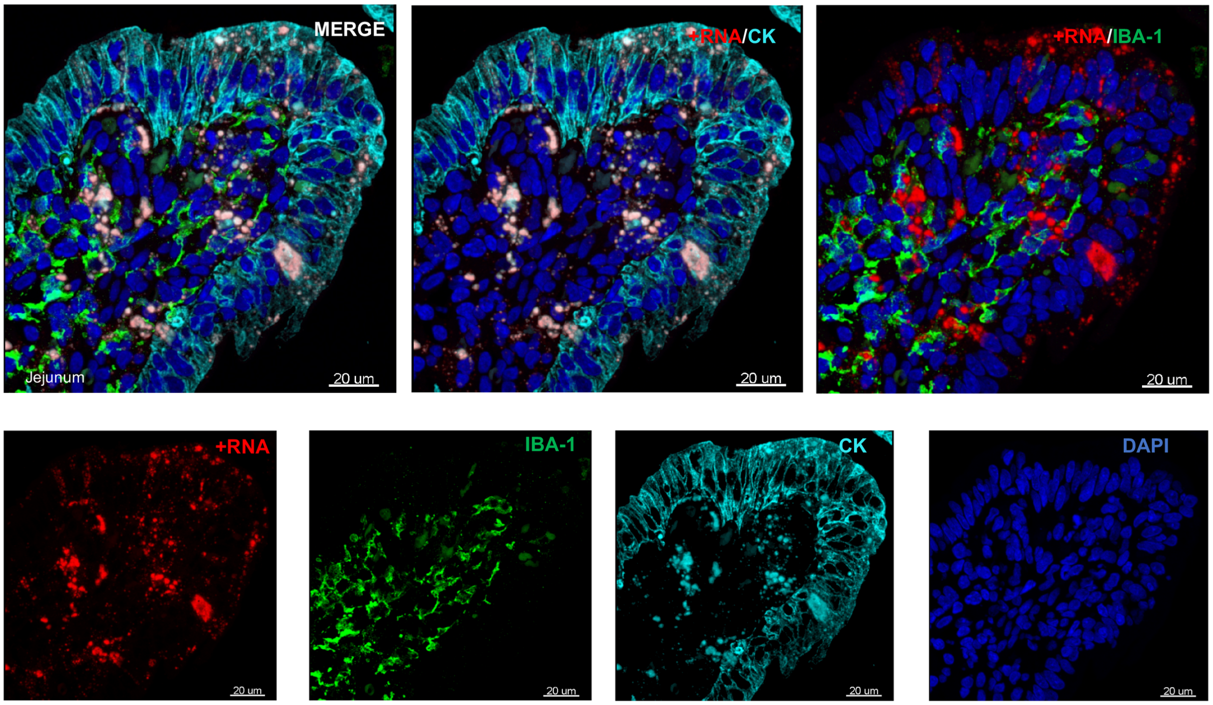

Supplementary Figure 11: Co-localization of cytokeratin with macrophage-associated norovirus positive strand RNA within an area of the lamina propria. The GT-1 biopsy was examined by confocal microscopy with four separate colored probes, positive sense RNA (red), IBA-1 (green), CK (cyan), and DAPI (blue). Merged image (first panel) shows co-localization of norovirus positive sense RNA with macrophage and cytokeratin in the lamina propria.

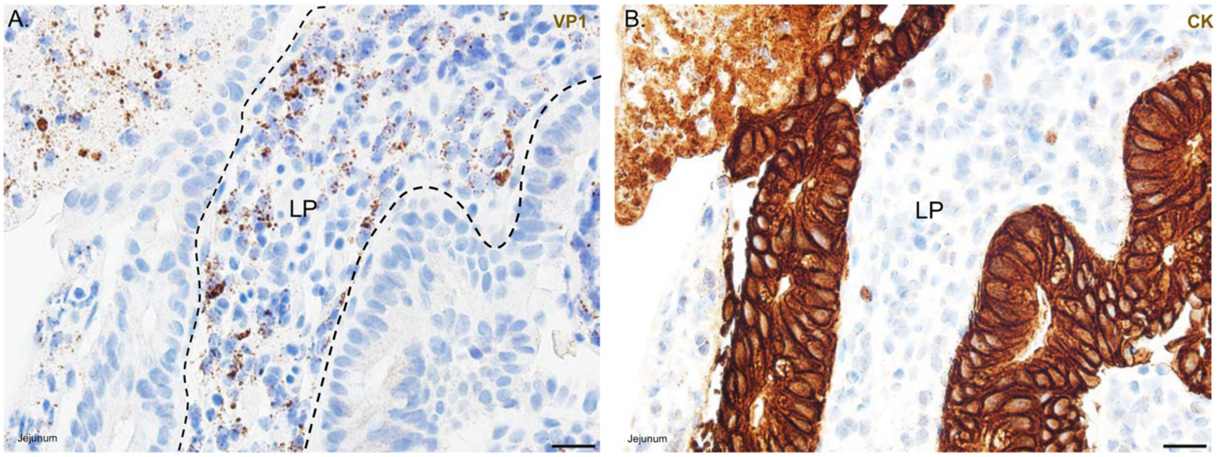

Supplementary Figure 12: Absence of co-localization between norovirus capsid protein VP1 and cytokeratin in an area of lamina propria. A. Chromogenic staining of an area of the jejunal biopsy of patient GT-1 probed only with monoclonal antibody TV19 for the detection of norovirus VP1 (brown) shows distribution of capsid protein within the lamina propria (LP). **B.** The same area shown in **A.** was probed only for expression of epithelial cell marker cytokeratin (CK) (brown). Magnification: 40x, scale bar represents 20μm.

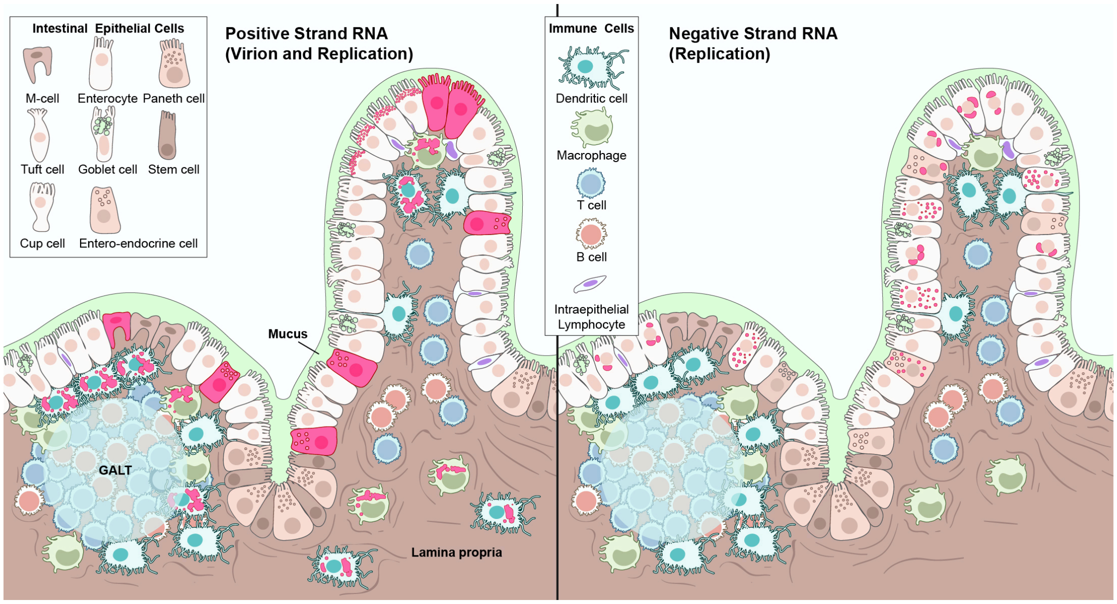

Supplementary Figure 13: Summary of the distribution patterns of human norovirus RNA observed in this study. Left panel (Positive Strand RNA) illustrates the three major patterns of positive strand RNA (shaded red) observed in intestinal tissue: apical distribution of punctate RNA signals toward the lumen in enterocyte-like epithelial cells, aggregated forms in myeloid-origin immune cells in the lamina propria, and intense cytoplasmic staining of contiguous or isolated epithelial cells. Right panel (Negative Strand RNA) shows the two major patterns of observed negative strand RNA signals (shaded red): discrete, sometimes perinuclear locations and a diffuse cytoplasmic distribution of dot-like signals, all predominantly in epithelial cells, including EECs. Drawing was created with Illustrator CC 2019 (Adobe, Inc.).

Supplementary Table 1: Norovirus-specific primers, probes, and RNA standards

Purpose	Name	Primer sequences (5’-3’)	Polarity	[1]Position
RT-qPCR	GII RNA Standard	AGCCAAUGUUCAGAUGGAUGAGAUUCUCAGAUCUGAGCACGUGGGAGGGCGAUCGCAAUCUGGCUCCCAGUUUU GUGAAUGAAGAUGGCGUCGAGU	+	5007-5102
	MGBGII_F	ATGTTYAGRTGGATGAGRTT	+	5012-5031
	MGBGII_R	ACGCCATCTTCATTCACA	-	5097-5080
	MGBGII_Probe	6FAM-TGGGAGGGCGATC-MGBNFQ	+	5048-5060
Diagnostic PCR and ORF1/2 Genotyping	GIIvpgF	GACTTCTGTGAGGAGGAGGA	+	2825-2844
	GIIpolF	GACCCAGCTGGTTGGTTTGG	+	4763-4782
	GII.4polF	CTCAGGCAAATGTACTGGACYA	+	4805-4826
	GII.3/6polF	CTCAGACAGWTGTACTGGACHA	+	4805-4826
	G2SKR	CCRCCNGCATRHCCRTTRTACAT	-	5389-5367
Subgenomic PCR and Capsid Sequencing	GII.P16polF	GCGATCGCAATCTGGCTCCCT	+	5055-5074
	3' TwinRockR	TTTTTTTTTTTTTTTAAAAGACACTAAAGAAAGAAAAAGA	-	7540-7564
	Pe-SydneyCap1F	GTGAATGAAGATGGCGTCGAG	+	5081-5101
	Pe-SydneyCap2F	AGCCAGGTCACTATGTTCCC	+	5490-5509
	Pe-SydneyCap3F	CTTCAGAGGAGATGTCACCCA	+	5939-5959
	PeSydneyORF3R	CAGCTGTAGAACCAAGTCTCGT	-	7253-7232
Restriction Digest Cloning (NS sera)	[2]NS5^{VPg}F	ATATA*CCATGG*GCAGCAGCCATCATCATCATCATCACAGCAGCGGCGGTAAGAAAGGGAAG	+	2630-2644
	[2]NS5^{VPg}R	GGTGCTCGAGT*GCGGCCGC***TTA**CTCAAAGTTGAGTTT	-	3014-3028
	[2]NS6^{Pro}F	ATATA*CCATGG*GCAGCAGCCATCATCATCATCATCACAGCAGCGGCGCCCCACCAAGCATC	+	3029-3043
	[2]NS6^{Pro}R	CGAGT*GCGGCCGC***TTA**TTCAAGTGTGGCTTCTCCCTCA	-	3550-3571
	[2]NS7^{Pol}F	ATATA*CCATGG*GCAGCAGCCATCATCATCATCATCACAGCAGCGGCGGGGGTGACAGTAAAG	+	3572-3587
	[2]NS7^{Pol}R	CGAGT*GCGGCCGC***TCA**CTCGACGCCATCTTCATTCACAAA	_	5078-5101

[1]Positions of primers in the norovirus genome designed for this study are numbered according to the genome sequence of norovirus GII.4 Sydney 2012 (JX459908).

[2]Restriction enzyme sites (NcoI or NotI) are italicized and engineered stop codons are shown in bold type. Norovirus gene-specific sequences are underlined.

Table description: Primers, probes, and a synthetic RNA standard used for norovirus molecular diagnostics, genotyping, genome quantification, and reagent development are summarized.

Supplementary Table 2. Commercial antibodies, stains, and conjugates

Marker/Antibody/Conjugate/Stain	Specificity	Source	Catalog #	Clone	Lot #	Dilution
Pancytokeratin (CK)	Epithelial Cells	Abcam	ab27988	AE1/AE3	GR108141-1	1:200
DC-SIGN (CD209)	Dendritic cells/macrophage	Abcam	ab5715	N/A[1]	GR3191400-1	1:500
IBA-1	Macrophage	Wako	019-19741	N/A	SAF5299	1:800 (IHC)
IBA-1	Macrophage	Abcam	ab107159	N/A	GR140190-10	1:1000 (IF)
CD20	B cells	Abcam	ab78237	EP459Y	GR246883-1	1:100 (IHC)
CD20	B cells	Abcam	ab64088	SP32	GR3260990-7	1:100 (IF)
CD3	T cells	Bio-Rad	MCA1477	CD3-12	0714R	1:600 (IHC) 1:400 (IF)
GP2	M Cells	Novus Biologicals	NBP1-86061	N/A	A115861	1:1000
CD4	T Cells	Abcam	ab133616	EPR6855	GR303505-4	1:500
CD103	Intraepithelial Lymphocytes	Novus Biologicals	NBP1-88142	N/A	C105230	1:50
Chromogranin A	Enteroendocrine cells	Biocare Medical	CM010	LK2H10 + PHE5	110104/51618	1:200 (IHC); 1:50 (IF)
Chromogranin A	Enteroendocrine cells	Abcam	ab15160	N/A	GR3229573-2	1:400
Choline Acetyltransferase	Tuft Cells	Abcam	ab178850	EPR16590	GR3230471-2	1:2000 (IHC); 1:500 (IF)
DAPI	Nuclei	Thermo Fisher	62248	N/A	SG2412512	1:1000
Unconjugated Rabbit Anti-Rat IgG Antibody, Mouse, Adsorbed	Rat IgG	Vector Laboratories	AI-4001	N/A	ZC0603	1:100
Goat anti-Rabbit IgG (H+L) Cross-Adsorbed Secondary Antibody, Alexa Fluor 488	Rabbit IgG	Thermo Fisher Scientific	A11008	N/A	1229706/1735088	1:500
DyLight 488 Horse Anti-Rabbit IgG Antibody	Rabbit IgG	Vector Laboratories	DI-1088	N/A	ZC1005	1:300
Biotinylated Horse Anti-Mouse IgG Antibody, Rat, Adsorbed	Mouse IgG	Vector Laboratories	BA-2001	N/A	Z0421	1:75
Biotinylated Horse Anti-Mouse IgG Antibody	Mouse IgG	Vector Laboratories	BA-2000	N/A	Y0907	1:200
Donkey anti-Mouse IgG (H+L) Highly Cross Adsorbed Secondary Antibody, Biotin	Mouse IgG	Invitrogen	A16021	N/A	44178011215	1:500
Donkey anti-Rabbit IgG (H+L) Highly Cross-Adsorbed Secondary Antibody, Alexa Fluor 488	Rabbit IgG	Invitrogen	A21206	N/A	913921	1:1000
Donkey anti-Rat IgG (H+L) Highly Cross-Adsorbed Secondary Antibody, Biotin	Rat IgG	Novus Biologicals	NBP1-75379	N/A	42-173-080614	1:500
Donkey anti-Goat IgG (H+L) Highly Cross Adsorbed Secondary Antibody, Biotin	Goat IgG	Thermo Fisher Scientific	A16009	N/A	42-150-070814	1:500
Streptavidin, Alexa Fluor™ 488 Conjugate	Biotin	Thermo Fisher Scientific	S32354	N/A	1571714	1:500
Streptavidin, Alexa Fluor™ 594 Conjugate	Biotin	Thermo Fisher Scientific	S32356	N/A	1661292	1:500
Streptavidin, Alexa Fluor™ 680 Conjugate	Biotin	Thermo Fisher Scientific	S32358	N/A	1969179	1:500

[1]N/A Not applicable

Table Description: The commercial source, clone number (if applicable), catalog number, lot number, and working dilution of antibodies, stains, and conjugates utilized in study.
