# Supplementary material for: Human norovirus targets enteroendocrine epithelial cells in the small intestine
Source: Nat Commun. 2020 Jun 2;11:2759. doi: 10.1038/s41467-020-16491-3 (PMC7265440; doi:10.1038/s41467-020-16491-3)
Supplement: Supplementary file 3 — Reporting Summary [file 41467_2020_16491_MOESM3_ESM.pdf]

nature research

Corresponding author(s): Kim Y. Green

Last updated by author(s): Apr 17, 2020

Reporting Summary

Nature Research wishes to improve the reproducibility of the work that we publish. This form provides structure for consistency and transparency in reporting. For further information on Nature Research policies, see Authors & Referees and the Editorial Policy Checklist .

Statistics

For all statistical analyses, confirm that the following items are present in the figure legend, table legend, main text, or Methods section.

n/a	Confirmed	
☐	☒	The exact sample size (n) for each experimental group/condition, given as a discrete number and unit of measurement
☐	☒	A statement on whether measurements were taken from distinct samples or whether the same sample was measured repeatedly
☒	☐	The statistical test(s) used AND whether they are one- or two-sided<br>*Only common tests should be described solely by name; describe more complex techniques in the Methods section.*
☒	☐	A description of all covariates tested
☒	☐	A description of any assumptions or corrections, such as tests of normality and adjustment for multiple comparisons
☒	☐	A full description of the statistical parameters including central tendency (e.g. means) or other basic estimates (e.g. regression coefficient) AND variation (e.g. standard deviation) or associated estimates of uncertainty (e.g. confidence intervals)
☒	☐	For null hypothesis testing, the test statistic (e.g. F, t, r) with confidence intervals, effect sizes, degrees of freedom and P value noted<br>*Give P values as exact values whenever suitable.*
☒	☐	For Bayesian analysis, information on the choice of priors and Markov chain Monte Carlo settings
☒	☐	For hierarchical and complex designs, identification of the appropriate level for tests and full reporting of outcomes
☒	☐	Estimates of effect sizes (e.g. Cohen's d, Pearson's r), indicating how they were calculated

Our web collection on statistics for biologists contains articles on many of the points above.

Software and code

Policy information about availability of computer code

Data collection	Leica Application Suite X (LAS X) (Leica Microsystems)
Data analysis	Imaris 9.2.1 (Bitplane, Oxford Instruments)<br>Photoshop CC 2019 (Adobe, Inc.)<br>Illustrator CC 2019 (Adobe, Inc.)

For manuscripts utilizing custom algorithms or software that are central to the research but not yet described in published literature, software must be made available to editors/reviewers. We strongly encourage code deposition in a community repository (e.g. GitHub). See the Nature Research guidelines for submitting code & software for further information.

Data

Policy information about availability of data

All manuscripts must include a data availability statement. This statement should provide the following information, where applicable:

- Accession codes, unique identifiers, or web links for publicly available datasets
- A list of figures that have associated raw data
- A description of any restrictions on data availability

Sequence data that were generated in this study have been deposited in GenBank with the primary accession code MN220720. https://www.ncbi.nlm.nih.gov/nuccore/MN220720

Microscopy imaging data are available from senior co-author Ian N. Moore upon reasonable request.

Field-specific reporting

Please select the one below that is the best fit for your research. If you are not sure, read the appropriate sections before making your selection.

- [x] Life sciences
- [ ] Behavioural & social sciences
- [ ] Ecological, evolutionary & environmental sciences

For a reference copy of the document with all sections, see nature.com/documents/nr-reporting-summary-flat.pdf

Life sciences study design

All studies must disclose on these points even when the disclosure is negative.

Sample size	Because available biopsy tissue from norovirus-infected patients is limited, we did not predetermine sample sizes. One acutely-ill norovirus patient, and two norovirus-negative patients receiving intestinal transplants at Medstar Georgetown University Hospital in Washington D.C. were studied in our original submission. Endoscopy of patients during acute diarrhea is rare, but in small intestinal transplant patients this procedure is required since there are no serological markers of organ rejection. This fact accounts for the small sample size of this study. It should be noted that this was an unusual opportunity to examine cell tropism in the presence of abundant virus since norovirus is more difficult to detect in tissue during chronic infection (which is the source of most biopsies for analysis). Following review, data from one additional pediatric Georgetown transplant recipient with norovirus and two patients from an NIH adult immunocompromised cohort were included in the revised manuscript to show that our discovery of EECs as target cells was reproducible beyond one patient. In total, 4/4 of immunocompromised patients with norovirus infection in this study were shown to have norovirus-positive EEC cells in their gut tissue. The two norovirus-negative patients in this study did not show evidence of norovirus-infected cells of any type.
Data exclusions	There were no data exclusions in this study.
Replication	Multiple sections of intestinal biopsies were examined independently 3-5 times with a battery of norovirus-specific and cell marker reagents. Data were reproducible among sections and tissues, and among the small number of patients in this study.
Randomization	Randomization was not possible in this study due to the small number of patients available for study.
Blinding	Blinding was not feasible due to the limited numbers and amounts of tissue specimens and knowledge of the clinical status of the patient at the time of biopsy analysis.

Reporting for specific materials, systems and methods

We require information from authors about some types of materials, experimental systems and methods used in many studies. Here, indicate whether each material, system or method listed is relevant to your study. If you are not sure if a list item applies to your research, read the appropriate section before selecting a response.

Materials & experimental systems

n/a	Involved in the study	
	x	Antibodies
x		Eukaryotic cell lines
x		Palaeontology
	x	Animals and other organisms
	x	Human research participants
x		Clinical data

Methods

n/a	Involved in the study	
x		ChIP-seq
x		Flow cytometry
x		MRI-based neuroimaging

Antibodies

Antibodies used	Commercially available antibodies: Pancytokeratin (CK), Abcam ab27988, clone AE1/AE3, Lot GR108141-1; DC-SIGN (CD209), Abcam ab5715, lot GR3191400-1; IBA-1, Wako 019-19741, lot SAF5299; CD20, Abcam ab78237, clone EP459Y, lot GR246883-1; CD3, Bio-Rad MCA1477, clone CD3-12, lot 0714R; GP2, Novus Biologicals NBP1-86061, lot A115861; CD4, Abcam ab133616, clone EPR6855, lot GR303505-4; CD103, Novus Biologicals NBP1-88142, lot C105230; Chromogranin A, Biocare Medical CM010, clones LK2H10+PHE5, lot 110104/51618; Chromogranin A, Abcam ab15160, lot GR3229573-2; Choline Acetyltransferase, Abcam ab178850, clone EPR16590, lot GR3230471-2; Unconjugated Rabbit Anti-Rat IgG Antibody, mouse, adsorbed, Vector Laboratories AI-4001, lot ZC0603; Goat anti-Rabbit IgG (H+L) Cross-Adsorbed Secondary Antibody, Alexa Fluor 488, Thermo Fisher Scientific A11008, lot 1229706/1735088; DyLight 488 Horse Anti-Rabbit IgG Antibody, Vector Laboratories DI-1088, lot ZC1005; Biotinylated Horse Anti-Mouse IgG Antibody, rat adsorbed Vector Laboratories BA-2001, lot Z0421; Biotinylated Horse Anti-Mouse IgG Antibody Vector Laboratories BA-2000, lot Y0907; Donkey anti-Mouse IgG (H+L) Highly Cross-Adsorbed Secondary Antibody, Biotin, Invitrogen A16021, lot 44178011215; Donkey anti-Rabbit IgG (H+L) Highly Cross-Adsorbed Secondary Antibody, Alexa Fluor™ 488, Invitrogen A21206, A913921; Streptavidin, Alexa Fluor™ 488 Conjugate, Thermo Fisher Scientific S32354, lot 1571714; Streptavidin, Alexa Fluor™ 594 Conjugate,Thermo Fisher Scientific S32356, lot 1661292; Streptavidin, Alexa Fluor™ 680 Conjugate, Thermo Fisher Scientific S32358, lot 1969179.

Norovirus-specific antibodies: VP1-specific monoclonal antibodies TV19 and 30A11 (referenced in paper); rabbit polyclonal antisera raised against nonstructural proteins NS5VPg, NS6Pro and NS7Pol described in present study

Validation	Commercial antibodies were validated by the manufacturer.<br>Norovirus-specific antibodies were validated at NIH with positive and negative recombinant norovirus antigen controls.

Animals and other organisms

Policy information about studies involving animals; ARRIVE guidelines recommended for reporting animal research

Laboratory animals	Antisera were raised in New Zealand white rabbits (mixed sex and 6 months of age) at Pocono Rabbit Farm and Laboratory, Inc, an approved NIH animal contractor and an AAALAC International accredited company.
Wild animals	No wild animals were used in this study.
Field-collected samples	No field samples were collected from animals in this study.
Ethics oversight	Ethics oversight was conducted by the Animal Care and Use Committee at Pocono Rabbit Farm and Laboratory, Inc.

Note that full information on the approval of the study protocol must also be provided in the manuscript.

Human research participants

Policy information about studies involving human research participants

Population characteristics	The Georgetown patients in this study (n=4) were enrolled in an intestinal transplant protocol for pediatric patients and all had received an intestinal transplant that was performed based on clinical criteria. Through standard clinical monitoring, two patients (designated GT-1 and GT-1211) were established to be infected with norovirus, and two (GT-4 and GT-5) were not infected with norovirus. Patient GT-1, the focus of this study, is male and Patient GT-1211, female.<br><br>NIH patient samples (n=2) were from adults enrolled in a samples study for the investigation of norovirus infection. Both patients (NIH 74 and NIH 76) were immunosuppressed post-stem cell transplantation. One was male and one female. These patients were selected for this study because they were confirmed to be norovirus-positive and tissue biopsy samples were available..
Recruitment	All patients and/or their legal representative who agreed to be placed on the national waitlist for intestinal transplant are offered the opportunity to participate in a bio-medical research protocol, Georgetown University IRB #2004-008 (""Clinical Investigation of Inflammatory Bowel Diseases, Intestinal Failure, and Transplantation"") and #2017-0365 (""Unleash the Potential of Immuno-Monitoring in Transplantation""), after the transplant has been performed. This research involves collection of extra biopsy material at the time of all protocol and ad hoc post- intestinal transplant endoscopies as well as other biological samples for placement in storage in the MedStar Georgetown Transplant Institute Biorepository for the express purpose of conducting bio-medical research at the discretion of participating clinical investigators at the Transplant Institute. Should patients or their legal representatives decline to participate in the above-referenced protocol, then no materials intended for biomedical research are collected. In this manuscript, biopsy material was utilized based on identification of norovirus infection in standard clinical practice. Additional biopsy material from consenting patients was also utilized for purposes of quality assurance. Patients enrolled in NIH LID 11-I-0109 ("Viral Infections in Healthy and Immunocompromised Patients") gave written consent to provide residual clinical samples obtained during the course of their care at NIH for other underlying conditions to the Laboratory of Infectious Diseases/NIAID to advance biomedical research on viral infections.
Ethics oversight	Georgetown University Institutional Review Board and the NIH Institutional Review Board

Note that full information on the approval of the study protocol must also be provided in the manuscript.
