# Supplementary material for: Human norovirus targets enteroendocrine epithelial cells in the small intestine
Source: Nat Commun. 2020 Jun 2;11:2759. doi: 10.1038/s41467-020-16491-3 (PMC7265440; doi:10.1038/s41467-020-16491-3)
Supplement: Supplementary file 4 — Description of Additional Supplementary Files [file 41467_2020_16491_MOESM4_ESM.pdf]

Description of Additional Supplementary Files

File Name: Supplementary Movie 1. Cytokeratin and negative sense norovirus RNA in subepithelial cell. Confocal z-stack images were acquired from GT-1 jejunum slices (shown in Fig. 2E) that were hybridized with the RNAscope probe specific for negative strand norovirus GII.4 RNA (red) and stained with anti-IBA-1 (green) and anti-cytokeratin antibodies (cyan). Nuclei of cells were stained with DAPI (blue). Using the Imaris 9.2.1 software (Bitplane), the z-stacks were reconstructed into a 3D image, and isosurfaces for each fluorescent channel were created. The 3D rendering shows localization of negative RNA and cytokeratin markers in close proximity, suggesting an epithelial origin for this cell.

File Name: Supplementary Movie 2. Chromogranin A (CgA) and negative sense norovirus RNA in epithelial cell. Confocal z-stack images were acquired from GT-1 jejunum slices (shown in Fig. 4C) stained with the RNAscope probe specific for negative strand norovirus GII.4 RNA (red) and anti-CgA antibodies (cyan). Images were processed using Imaris software similar to Supplementary Movie 1. The 3D rendering shows expression of both chromogranin A and norovirus negative strand RNA markers within the same cell.

File Name: Supplementary Movie 3. Relationship between chromogranin A (CgA), negative sense norovirus RNA, and VP1 in a single cell. Confocal z-stack images were acquired and stained with the RNAscope probe specific for negative strand norovirus GII.4 RNA (red), monoclonal antibody 30A11 to detect VP1 (cyan), and anti-CgA antibodies (green). Images

were processed using Imaris software similar to Supplementary Movie 1. The 3D rendering shows expression of CgA, VP1, and norovirus negative strand RNA markers within the same cell.

File Name: Supplementary Movie 4. Relationship between norovirus capsid protein VP1 and myeloid cell marker DC-SIGN. Confocal z-stack images were acquired from GT-1 jejunal slices stained with broadly reactive VP1-specific mAb TV19 (red) and anti-DC-SIGN antibodies (green). Images were processed similar to Movie 1 with one modification – 3D rendering was performed in the image normal shading mode of Imaris. The 3D rendering shows that DC-SIGN positive cells near the epithelial layer and within the lamina propria contain norovirus capsid antigen.

File Name: Supplementary Movie 5. Relationship between norovirus capsid protein VP1 and CD4-positive T cell. Confocal z-stack images were acquired from GT-1 jejunum slices stained with mAb TV19 (red) and anti-CD4 antibodies recognizing the CD4 surface glycoprotein (green). Fluorescent imaging indicates presence of CD4-expressing cell in proximity to norovirus VP1 antigen, however, 3D rendering of T cell shows viral antigen outside the cell, although in close contact. Images were processed using Imaris software similar to Supplementary Movie 1.

File Name: Supplementary Movie 6. Norovirus negative sense RNA and lymphoid immune cell markers. Confocal z-stack images were acquired from GT-1 jejunum slices that were hybridized with the RNAscope probe specific for negative strand norovirus GII.4 RNA (white) and stained with antibodies specific for CD3-positive T cells (magenta) and CD20-positive B cells (green). Images were processed using Imaris software similar to Supplementary Movie 1. The 3D

rendering shows localization of negative sense norovirus RNA in cells other than T- and B-cells in the surrounding tissue.
